# Editorial: Molecular mechanisms of glutamatergic synapse function and dysfunction, volume II

**DOI:** 10.3389/fnmol.2025.1618161

**Published:** 2025-05-20

**Authors:** Dhrubajyoti Chowdhury, Jonathan G. Murphy

**Affiliations:** ^1^School of Science, Gandhi Institute of Technology and Management (GITAM), Vishakhapatnam, India; ^2^St. Jude Children's Research Hospital, Memphis, TN, United States

**Keywords:** glutamate, synapse, PSD, plasticity, excitatory synapse, AMPARs, kainate receptors, NMDARs

Synapses are the building blocks of neural circuits and represent the means for relay of peripheral sensory information to the neocortex and between brain regions. Optimal brain function requires the proper development, maturation and maintenance of synapses. The excitatory synapses of the mammalian central nervous system rely on glutamate as a neurotransmitter. The ionotropic glutamate receptors (iGluRs) and their associated signaling complexes control the postsynaptic response to presynaptic release of glutamate and, therefore, remain under tight regulation by a variety of molecular mechanisms. Regulation of the synaptic proteome is achieved largely by protein-protein interactions and post-translational modifications. Identifying such mechanisms and understanding how external factors and experiences can influence them are essential for deciphering animal behavior and the etiology of disease. Importantly, experience-dependent changes in glutamatergic synaptic transmission underlie plasticity in brain function associated with learning and memory. Not surprisingly, dysregulation of glutamatergic synapses precedes the onset of symptoms across a wide spectrum of neurological and psychiatric disorders ranging from stroke and movement disorders to intellectual disability and dementia. Therefore, studies into molecular mechanisms of synapse biology not only provide fundamental insights into the functional core of the nervous system but also lay the foundation for the development of novel therapeutic approaches. This Research Topic comprises five articles that explore the latest discoveries in the molecular pathways involved in synapse function and dysfunction with a particular focus on glutamatergic synapses.

The input-selective molecular composition of excitatory synapses is only beginning to be understood thanks to recent technological advances. Robinson et al. used expansion microscopy to image labeled glutamatergic synaptic proteins in the mouse cerebellar cortex at single-synapse resolution. This study used synapse-specific markers to map glutamate receptors and postsynaptic scaffolding proteins, including PSD-95, SAPAP1, Shank1/2/3, and the GTPase activating protein SynGAP to define the molecular identity of several classes of cerebellar synapse types. The authors identified synapse-selective distributions of AMPA/NMDA receptors, Shank and SynGAP across multiple synapse types. Interestingly, NMDA receptors and SynGAP were preferentially localized to extrasynaptic clusters and adherens junctions in glomeruli of the granule cell layer, suggesting differential neurotransmitter receptor signaling. This work presents a synapse-specific molecular framework that will aid in linking cerebellar circuit function with synapse types.

Post-translational modifications (PTMs) of synaptic proteins play critical roles in activity-dependent tuning of excitatory synapse function. Being the primary drivers of synapse function, glutamate receptors are obvious substrates for PTMs that are known to control receptor trafficking. Kainate receptors (KARs) are a class of glutamate receptors that are subject to a diverse array of activity-dependent PTMs. Yucel et al. investigates the sequence, coordination, and interplay between palmitoylation, phosphorylation, and SUMOylation-dependent regulation of GluK2-containing KARs. This study uncovers that basal GluK2 palmitoylation stabilizes KAR surface expression. Moreover, depalmitoylation of surface KARs is a pre-requisite for agonist-induced endocytosis. Agonist-induced depalmitoylation promotes PKC phosphorylation (S868) and SUMOylation (K886) of GluK2, driving endocytosis. Non-palmitoylatable mutants exhibit reduced surface expression and impaired internalization, revealing coordinated PTM regulation of KAR trafficking.

The surface trafficking of heterotetrameric AMPARs are dynamically regulated by activity and pathological agents acting at the synapse. Real-time quantitative measurements of receptor redistribution between the cell surface and intracellular membranes are challenging but necessary to detect such dynamic regulation. The study by Prinkey et al. uses fluorescence lifetime imaging (FLIM) to measure surface trafficking of superecliptic pHluorin (SEP)-tagged AMPARs in living neurons. Subunit-specific effects of chronic amyloid-beta (Aβ) exposure were revealed. While GluA1/GluA2 receptors show higher intracellular accumulation, GluA1/GluA3 receptors show reduced surface and intracellular levels. This suggests that subunit composition dictates the fate of internalized AMPARs, possibly through specific interactions with the endolysosomal machinery. Interestingly, overexpression of Protein phosphatase 1 (PP1) mimics Aβ-induced synaptic depression by driving GluA1 endocytosis, confirming the role of phosphorylation in AMPAR trafficking. Simultaneous quantitative measurement of surface/internalized AMPARs in live cells without any normalization is a useful technological advancement for the field.

Apart from the receptors, the postsynaptic compartment is packed with an enormous number of proteins that assemble into local macromolecular complexes that mediate signal transduction. One such signaling complex is formed by the Traf and Nck interacting kinase (TNIK) that Jiang et al. characterized for its role in synapse development. Using patient-derived iPSC neurons, this study investigates how TNIK mutations linked to neurodevelopmental disorders (NDDs) disrupt postsynaptic density (PSD) signaling. Truncation (pArg180^*^) and kinase-dead (K54R) mutations increased spike frequency and impaired PSD structural components and MAP Kinase pathways in immature synapses. Phosphoproteomic analysis of mutant PSDs mapped changes to key functional classes of proteins, indicating a pivotal role for TNIK in synapse development. In addition, TNIK's interactome in immature synapses partially overlaps with mature rodent synapses, highlighting age-specific activities of this PSD signaling hub.

The article by Dong et al. presents a comprehensive review of recent studies identifying underlying mechanisms of NMDAR hypofunction in several disease states, including autism, intellectual disability, schizophrenia, age-related cognitive decline, and anti-NMDAR encephalitis. The authors discuss how NMDAR hypofunction serves as a convergence point for diverse pathological pathways in a variety of neurological disorders. NMDARs are highly interesting targets for developing therapeutics. Endogenous co-agonists (e.g., D-serine) and positive allosteric modulators, such as polyamines, are being extensively tested for enhancing NMDAR function in pre-clinical and clinical studies. While the precise mechanisms and treatments of NMDAR hypofunction remain obscure, we hope this article will stimulate new ideas for further exploration in this field for comprehending the pathogenesis of cognitive disorders and discovering novel therapeutic approaches.

